# Assessment of the Modified Rankin Scale in Electronic Health Records With a Fine-Tuned Large Language Model: Development and Internal Validation

**DOI:** 10.2196/82607

**Published:** 2026-02-25

**Authors:** Luis Silva, Marcus Milani, Sohum Bindra, Salman Ikramuddin, Megan Tessmer, Kaylee Frederickson, Abhigyan Datta, Halil Ergen, Alex Stangebye, Dawson Cooper, Kompal Kumar, Jeremy Yeung, Kamakshi Lakshminarayan, Christopher Streib

**Affiliations:** 1 Department of Neurology, University of Florida, 1600 SW Archer Road, Gainesville, FL, 32608, United States, 1 7633373761; 2 Department of Neurology, University of Minnesota, Minneapolis, MN, United States; 3 Department of Physical Therapy, Gaziantep University, Gaziantep, Turkey; 4 Department of Physical Therapy, M Health Fairview, Minneapolis, MN, United States

**Keywords:** stroke, modified Rankin scale, artificial intelligence, large language model, machine learning, electronic health record

## Abstract

**Background:**

The modified Rankin scale (mRS) is an important metric in stroke research, often used as a primary outcome in clinical trials and observational studies. The mRS can be assessed retrospectively from electronic health records (EHRs), but this process is labor-intensive and prone to interrater variability. Large language models (LLMs) have demonstrated potential in automating text classification.

**Objective:**

We aimed to create a fine-tuned LLM that can analyze EHR text and classify mRS scores for clinical and research applications.

**Methods:**

We performed a retrospective cohort study of patients admitted to a specialist stroke neurology service at a large academic hospital system between August 2020 and June 2023. Each patient’s medical record was reviewed at two time points: (1) at hospital discharge and (2) approximately 90 days post discharge. Two independent researchers assigned an mRS score at each time point. Two separate models were trained on EHR passages with corresponding mRS scores as labeled outcomes: (1) a multiclass model to classify all seven mRS scores and (2) a binary model to classify functional independence (mRS scores 0‐2) versus non-independence (mRS scores 3‐6). Four-fold cross-validation was conducted using accuracy and the Cohen κ as model performance metrics.

**Results:**

A total of 2290 EHR passages with corresponding mRS scores were included in model training. The multiclass model—considering all seven scores of the mRS—attained an accuracy of 77% and a weighted Cohen κ of 0.92. Class-specific accuracy was the highest for mRS score 4 (90%) and the lowest for mRS score 2 (28%). The binary model—considering only functional independence versus non-independence—attained an accuracy of 92% and a Cohen κ of 0.84.

**Conclusions:**

Our findings demonstrate that LLMs can be successfully trained to determine mRS scores through EHR text analysis; however, improving discrimination between intermediate scores is required.

## Introduction

The modified Rankin scale (mRS) is an important metric in stroke research, often used as a primary outcome in clinical trials and observational studies [1,2]. The mRS is scored from 0 (no symptoms) to 6 (death), with higher scores indicating greater disability. It is determined based on a patient’s stroke deficits and ability to perform daily activities [3]. It has also been used in pivotal stroke trials as a binary outcome comparing functional independence (scores 0‐2) versus non-independence (scores 3‐6) [4]. In most instances, trained clinicians or researchers collect the mRS score in real time. Alternatively, it can be assessed retrospectively from electronic health records (EHRs), but this process is labor-intensive and prone to interrater variability [5-8]. These limitations constrain stroke research by making it dependent on the availability of trained research staff and prevents the use of existing clinical databases for research.

Large language models (LLMs), such as GPT-4 (OpenAI), are deep learning–based models that perform well in text classification and generation [9,10]. Their use in medical research is expanding, with notable examples in neuroscience. GPT-4 has demonstrated 84% accuracy in localizing neurologic lesions and has also achieved a passing grade on the American Board of Psychiatry and Neurology examination [11,12]. However, GPT-4 performed poorly when assessing the Glasgow Coma Scale, the Intracranial Hemorrhage score, and the Hunt and Hess classifications [13]. These studies did not involve fine-tuning, a process in which the base model is trained on task-specific data to enhance performance. Classification of mRS scores from EHR text has been previously studied by Fernandez et al [14] who, using a non-LLM model, achieved 59% accuracy, limiting its practical application in both clinical and research settings.

We hypothesize that a fine-tuned LLM can analyze EHR text from inpatient and outpatient settings and classify mRS scores for clinical and research applications. We aimed to develop a tool capable of streamlining observational stroke research and reducing reliance on trained research staff.

## Methods

### Study Design

We performed a retrospective cohort study of patients evaluated by a stroke neurology service at a large academic hospital system between August 2020 and June 2023. Each patient’s medical record was reviewed at two time points: (1) at hospital discharge and (2) approximately 90 days post discharge, with follow-up notes selected within a window of 30 to 120 days. To minimize variability in follow-up timing, researchers were instructed to evaluate clinical notes recorded as close as possible to 90 days post discharge. If no appropriate notes were available, they gradually expanded the search window in both directions, extending to a final range of 30 to 120 days post discharge.

At each time point, two independent researchers—trained and certified in mRS assessment—assigned an mRS score. The mRS scoring followed the Rankin Focused Assessment, a structured checklist that standardizes patient evaluation through a question-answer format. Researchers assigned scores by answering predefined questions about functional status and stroke-related deficits [15]. Discrepancies in mRS scoring were resolved through discussion. If no consensus was reached, a third reviewer adjudicated the case.

**Figure 1.**
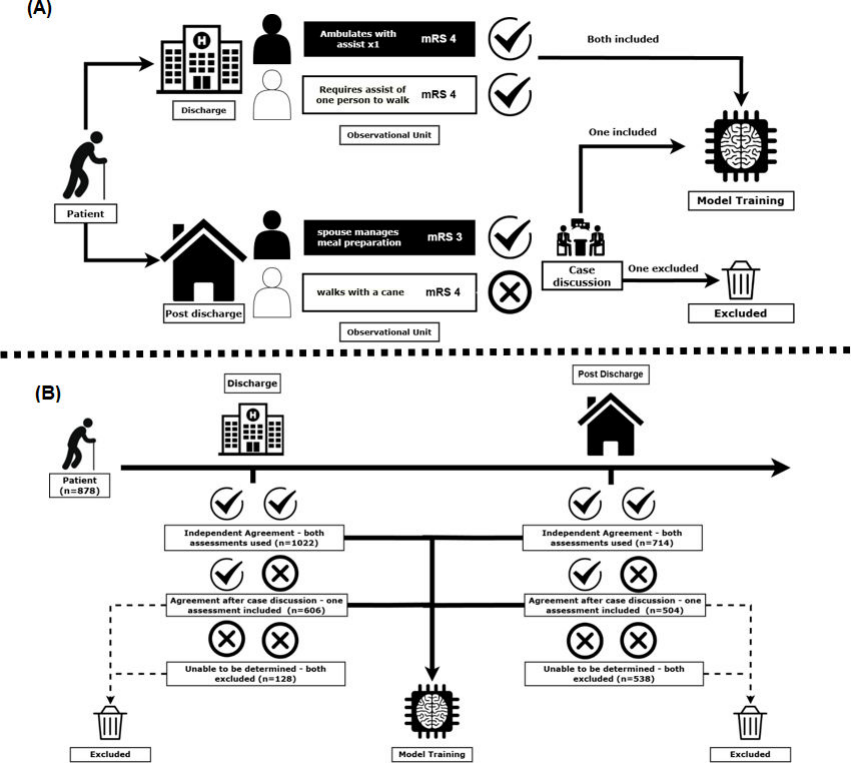
(A) Illustrative example of the data inclusion and exclusion process for each potential observational unit of a hypothetical patient. (B) Actual data inclusion and exclusion for large language model (LLM) training within our study cohort. mRS: modified Rankin scale.

Additionally, researchers identified and collected one corresponding EHR passage from the clinical note deemed critical for determining the mRS score. EHR passages were short, verbatim, and continuous text excerpts from a single clinical note. These typically originated from a physician, nursing, occupational therapy, or physical therapy note (Table 1). To prevent data leakage, direct mentions of mRS scores could not be included. EHR passages collected by researchers were used in their original form, without additional preprocessing or normalization beyond tokenization. This process generated the study’s observational unit: a paired EHR passage and mRS score. Because multiple, distinct EHR passages can support a single mRS score, each patient could contribute up to four EHR passage–mRS score pairs to the dataset: two from the discharge summary and two from the follow-up visit (one from each researcher at each time point). If the two researchers initially disagreed on the mRS score before reaching consensus, only the correctly adjudicated mRS score–EHR passage was included in the dataset. If a consensus could not be reached on the mRS score, both observational units were excluded from the analysis. Figure 1A illustrates EHR text collection, mRS scoring, and data inclusion decisions for model training.

**Table 1.** Model training and evaluation data characteristics.

Characteristics of the observational units	Discharge (n=1325)	Post discharge (n=966)
Age (y), median (IQR)	71 (60-81)	70 (59-80)
Stroke type, n (%)		
Ischemic	1116 (84.2)	844 (87.4)
Hemorrhagic	133 (10.0)	79 (8.2)
Not a stroke	75 (5.7)	42 (4.3)
Days from hospitalization to when the original note was written, median (IQR)	1 (1-3)	79 (56-103)
Profession of the provider whose text was used, n (%)		
Nurse	151 (11.4)	128 (13.3)
Occupational therapist	321 (24.2)	79 (8.2)
Physician	361 (27.2)	520 (53.8)
Physical therapist	398 (30.0)	79 (8.2)
Other (eg, physician assistant, speech-language pathologist, or social worker)	84 (6.3)	155 (16.0)
mRS^a^ score, n (%)		
0	232 (17.5)	250 (25.9)
1	176 (13.3)	264 (27.3)
2	66 (5.0)	129 (13.4)
3	183 (13.8)	166 (17.2)
4	463 (34.9)	80 (8.3)
5	132 (10.0)	43 (4.5)
6	73 (5.5)	34 (3.5)
Dichotomous mRS score, n (%)		
Functionally independent (mRS scores 0-2)	474 (36.7)	643 (67.6)
Functionally dependent (mRS scores 3-6)	851 (62.3)	323 (32.4)
Confidence score, n (%)		
5 - Answers a specific question of the Rankin Focused Assessment	442 (33.4)	206 (21.3)
4 - Does not answer a specific question, but almost certain	596 (45.0)	338 (35.0)
3 - Between two scores	258 (19.5)	324 (33.5)
2 - Between three scores	30 (2.3)	81 (8.4)
1 - Guess	2 (0.2)	12 (1.2)

a mRS: modified Rankin scale.

### Model Development

To develop our model, we used GatorTron-Base [16], an existing clinical LLM containing 345 million parameters, which include deidentified clinical notes from the University of Florida Health System, deidentified clinical notes from MIMIC-III, and peer-reviewed medical research. This allows it to capture the nuances of EHR text, making it suitable for our research. This model was chosen based on its performance in early study data, compared with other publicly available EHR-based models. Details of this analysis are provided in Multimedia Appendix 1; the TRIPOD (Transparent Reporting of a Multivariable Prediction Model for individual Prognosis or Diagnosis) checklist is provided in Checklist 1. All performance metrics were calculated as an average of four-fold cross-validation using 1000 bootstrapped samples to ensure reliable performance metrics. We fully fine-tuned GatorTron on our dataset to classify patients’ mRS scores based on the paired EHR passage. Fine-tuning is the process of further training a pretrained language model on a specific task—in this case, classifying mRS scores from EHR passages—to enable the model to adapt its general language understanding to the nuances of functional outcome assessment in stroke [17].

Two separate models were trained: (1) a multiclass model to classify all seven mRS scores and (2) a binary model to classify functional independence (mRS scores 0‐2) versus non-independence (mRS scores 3‐6). Four-fold cross-validation was conducted, using accuracy and unweighted and weighted Cohen κ as performance metrics.

Significant class imbalance was noted prior to model training; classes 2, 5, and 6 had less than 10% of the overall dataset, as seen in Table 1. Given this class imbalance, each mRS score was assigned training weights inversely proportional to its relative frequency in the overall data during model training to prevent overfitting.

### Ethical Considerations

This study was approved by the University of Minnesota—Twin Cities Institutional Review Board (STUDY0001939). The requirement for informed consent was waived by the institutional review board. All study data were deidentified prior to analysis. Data were stored and managed using REDCap (Research Electronic Data Capture; Vanderbilt University).

## Results

A total of 878 patients were evaluated, contributing 2290 observational units (EHR passage–mRS score pairs) to LLM training. Observational units were distributed across two stages: discharge (n=1325) and 90 days post discharge (n=966). This represented 75.1% (1325/1765) of the potential observational units at discharge and 54.7% (966/1765) of the potential observational units from the post-discharge period. Figure 1B details the process of the EHR passage–mRS score pairs’ inclusion and exclusion at each time point. The demographics were similar between groups, with a median (IQR) age of 71 (60‐81) years versus 70 (59‐80) years, ischemic stroke proportions of 84.2% (1116/1325) versus 87.4% (844/966), and hemorrhagic stroke proportions of 10.0% (133/1325) versus 8.2% (79/966) in the discharge and post-discharge groups, respectively (Table 1). The median (IQR) time from hospitalization to discharge was 1 (1‐3) day and that from hospitalization to follow-up was 79 (56‐103) days.

**Figure 2.**
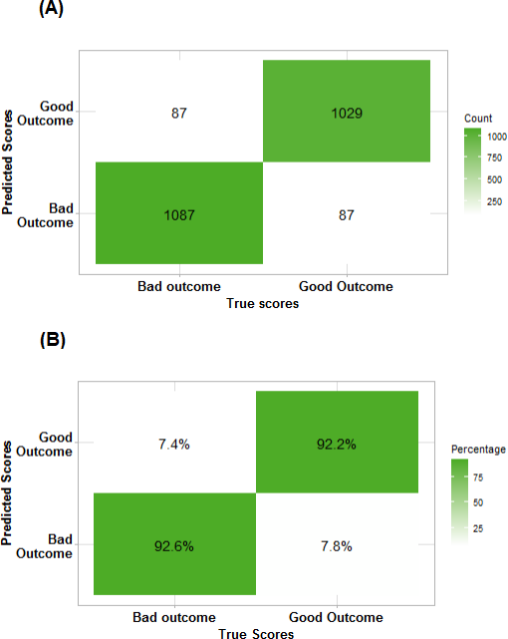
Confusion matrix of the binomial classification model as (A) absolute counts and (B) class accuracy. The model showed high accuracy for both classes.

**Figure 3.**
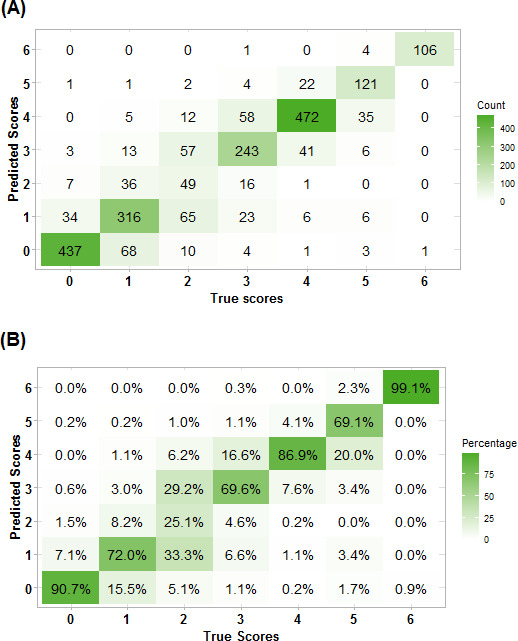
Confusion matrix of the multiclass classification model as (A) absolute counts and (B) class accuracy. These patterns suggest less certainty in classes with moderate disability.

The two-class model, which combined the mRS scores into two categories (mRS scores 0‐2 vs mRS scores 3‐6), had an accuracy of 92% (95% CI 91%‐93%) and a Cohen κ of 0.85 (95% CI 0.83‐0.87); the model’s confusion matrix is shown in Figure 2. The multiclass model, which included all seven categories of the mRS, achieved an accuracy of 77% (95% CI 76%‐79%), a Cohen κ of 0.71 (95% CI 0.69‐0.73), and a weighted κ of 0.92 (95% CI 0.90‐0.94). Figure 3 represents the confusion matrix for this model and shows that the highest classification accuracy occurred for scores of 0 (90%) and 6 (99%). Misclassification was more common among intermediate scores, particularly scores of 2, where class accuracy was limited to 25%.

Detailed performance metrics, including sensitivity (recall), specificity, positive predictive value (precision), and area under the receiver operating characteristic curve, are summarized in Multimedia Appendix 1.

**Figure 4.**
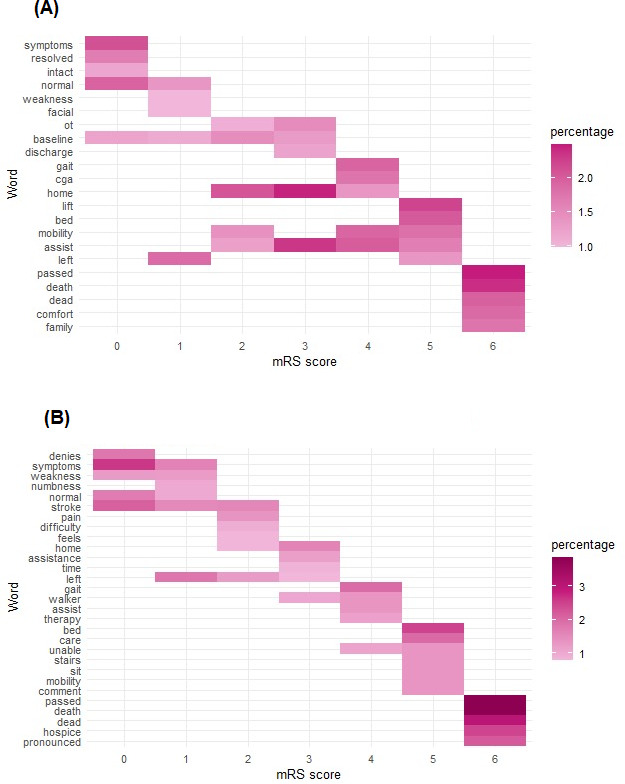
Heatmap of the most common words extracted in the electronic health record (EHR) passage for each class at (A) discharge and (B) long-term follow-up. Keywords associated with extreme classes, modified Rankin scale (mRS) scores 0‐1, and mRS scores 5‐6 had higher frequency of common words.

Greater variability was observed in the most common words for intermediate mRS scores versus extreme scores, which complicated their classification. This finding is illustrated by Figure 4, a heatmap of the most common words in each mRS strata.

## Discussion

### Principal Findings in the Context of Previous Research

The mRS has become a standard outcome measure in stroke clinical trials due to its ability to capture significant levels of disability while remaining intuitive and less time-intensive to ascertain compared to more detailed metrics [15,18]. Automated tools capable of deriving mRS scores from EHRs could streamline clinical research and potentially enable large-scale analyses using existing databases that lack mRS outcome data.

Our multiclass model demonstrated an accuracy of 77%, marking a significant improvement over previously published machine learning models for mRS determination from the EHR, which reported an accuracy of 59% [14]. Furthermore, our model also had a Cohen κ of 0.71 and a weighted κ of 0.92. The weighted κ, which penalizes larger classification errors more heavily than smaller ones, further underscores the model’s performance by indicating that most errors are near misses rather than substantial misclassifications. In a systematic review of stroke trials, human raters classified mRS scores with an average agreement accuracy of 73%, unweighted κ of 0.62, and weighted κ of 0.87 [6]. This suggests our model performs comparably to human raters and could assist in real-world applications.

Our two-class model achieved an accuracy of 92% in differentiating patients who achieved functional independence (mRS scores 0‐2) from those with functional dependence (mRS scores 3‐6). This demonstrates that reducing the number of classes can significantly improve model performance, a key consideration depending on application.

We propose that LLMs could reduce the manual chart review burden in clinical trials and registries. For example, a current standard research practice is to have two researchers with mRS certification independently evaluate each case and then assess agreement, with further adjudication by a third researcher for ambiguous cases. Partnering a certified researcher with a validated LLM for the initial mRS determination would improve operational efficiency. Further advancements in LLMs could enable scalable, consistent mRS extraction from unstructured clinical text. High-end use case examples might include clinical decision support systems or integration into learning health systems to inform iterative, data-driven care pathways.

Although our findings hint at the vast potential of LLMs in clinical research and clinical care applications, our study also highlights important current limitations. During LLM training, class imbalance necessitated weight classes inversely proportional to frequency. Although this may improve accuracy for less frequent mRS scores, such as mRS scores of 5 and 6, the classification of mRS scores of 2 remained challenging. Misclassification, especially between mRS scores of 2 and 3 (which distinguishes patients with functional independence vs non-independence), could be problematic in both research (eg, incorrect conclusions regarding efficacy) and clinical (eg, inappropriate discharge disposition) applications. However, this finding may be more reflective of the limitations inherent in the mRS than our LLM. In a study of 7374 patients, mRS scores of 0‐2 showed narrow variability in Longshi scale and Barthel Index scores, whereas mRS scores of 2‐4 exhibited much broader variability, suggesting poorer differentiation of moderate and severe disability states [19]. In future applications, hierarchical classification of patients using a pipeline of our binary model followed by our multiclass model could be used to mitigate the misclassification of scores 2 and 3. We did not implement it here due to limitations in dataset size. In addition, our dataset only included cases where mRS score consensus was achieved and excluded cases where EHR notes were ambiguous. While this design helped maintain a reliable dataset, excluding cases with poor documentation compromises the generalizability of our model to real-world applications.

### Conclusions

While promising, these models should currently be viewed as research-support tools to assist data abstraction rather than as stand-alone clinical instruments. Continued validation on multicenter datasets will be essential before clinical deployment. With further advancements of artificial intelligence in stroke research, fully automating mRS scoring from unstructured clinical text could integrate real-time outcome metrics into learning health systems.

Our findings, though preliminary, support the continued validation, investigation, and integration of LLMs into medical research and clinical care. They demonstrate that a fine-tuned LLM using EHR passages accurately classified 77% of mRS scores in the multiclass model (mRS scores 0‐6) and 92% of mRS scores in the two-class model (stratifying mRS scores 0‐2 vs mRS scores 3‐6). The multiclass model had the most difficulty differentiating between mRS scores of 2 and 3.

## Supplementary material

10.2196/82607 Multimedia Appendix 1 Model information and detailed model results.

10.2196/82607 Checklist 1 The TRIPOD checklist.

## Abbreviations

EHR: electronic health record
LLM: large language model
mRS: modified Rankin scale
REDCap: Research Electronic Data Capture
TRIPOD: Transparent Reporting of a Multivariable Prediction Model for individual Prognosis or Diagnosis
