# Supplementary material for: Assessment of the Modified Rankin Scale in Electronic Health Records With a Fine-Tuned Large Language Model: Development and Internal Validation
Source: JMIR AI. 2026 Feb 25;5:e82607. doi: 10.2196/82607 (PMC12935414; doi:10.2196/82607)
Supplement: Multimedia Appendix 1 [file ai-v5-e82607-s001.docx]

Multimedia Appendix 1

1. Base model determination

We used preliminary data to determine the best pre-trained model on EHR data. Binary classification—functional independence (mRS 0–2) versus non-independence (mRS 3–6)—was chosen as the outcome for comparison. The tested models were those available on the University of Minnesota HIPAA-compliant servers at the time of the preliminary analysis. All models chosen had to be at least partially trained in real EHR. Confusion matrices with absolute counts for each model are summarized below:

**BioClinicalBERT**

|  | True Good | True Bad |
| --- | --- | --- |
| Predict Good | **31** | **11** |
| Predict Bad | **19** | **17** |
| Accuracy = 61% |  |  |

**COrE Model**

|  | True Good | True Bad |
| --- | --- | --- |
| Predict Good | **38** | **12** |
| Predict Bad | **12** | **16** |
| Accuracy = 69% |  |  |

**GatorTron**

|  | True Good | True Bad |
| --- | --- | --- |
| Predict Good | **42** | **15** |
| Predict Bad | **8** | **13** |
| Accuracy = 71% |  |  |

Based on this preliminary analysis, GatorTron demonstrated the highest overall accuracy and the highest accuracy in functional independence determination. This was the rationale for choosing the model.

1. **Model information**

Multiclass model:

Base model: <https://huggingface.co/UFNLP/gatortron-base>

GPU used: NVIDIA A100 Tensor Core GPU (x1)

Time to complete training: 34 hours

Training and evaluation performed in HIPAA-compliant servers

Learning Rate: 1e-7

Batch size (training and evaluation): 32

Epochs: 200

Performance confidence intervals calculated based on 1,000 bootstrapped samples

Figure S1. Multiclass model Training/validation loss curves


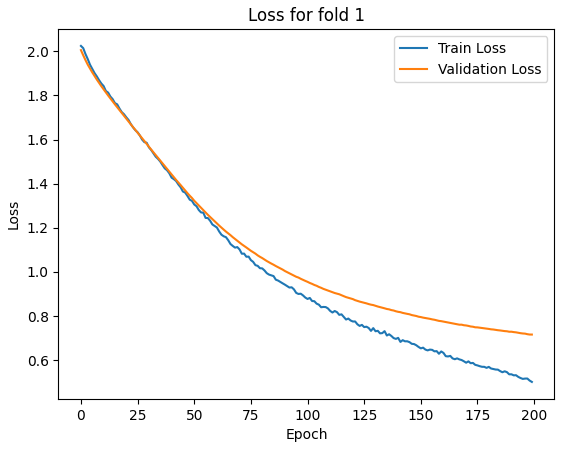

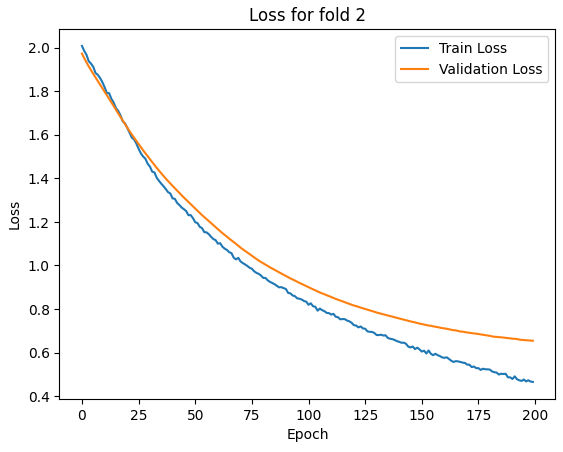

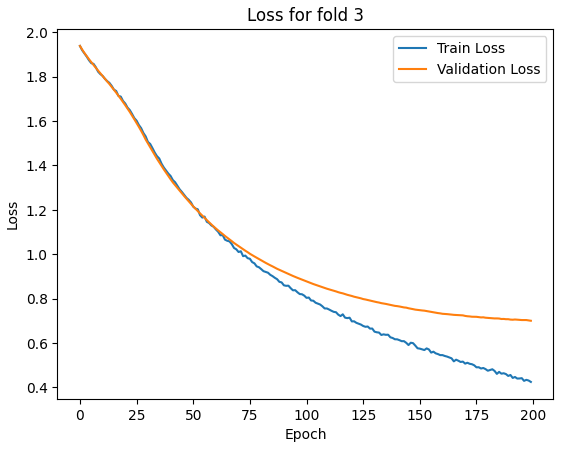

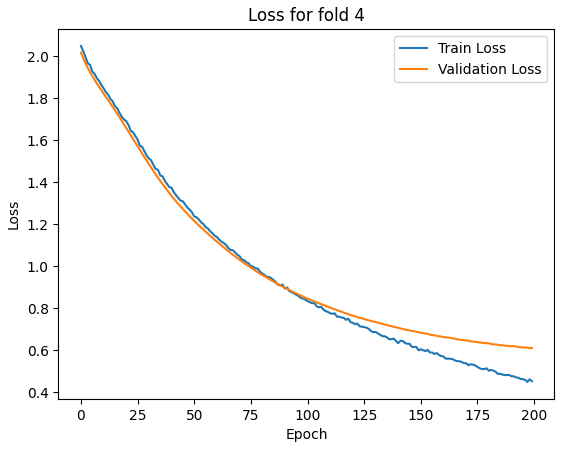


Figure S2. Calibration plot of the multiclass model


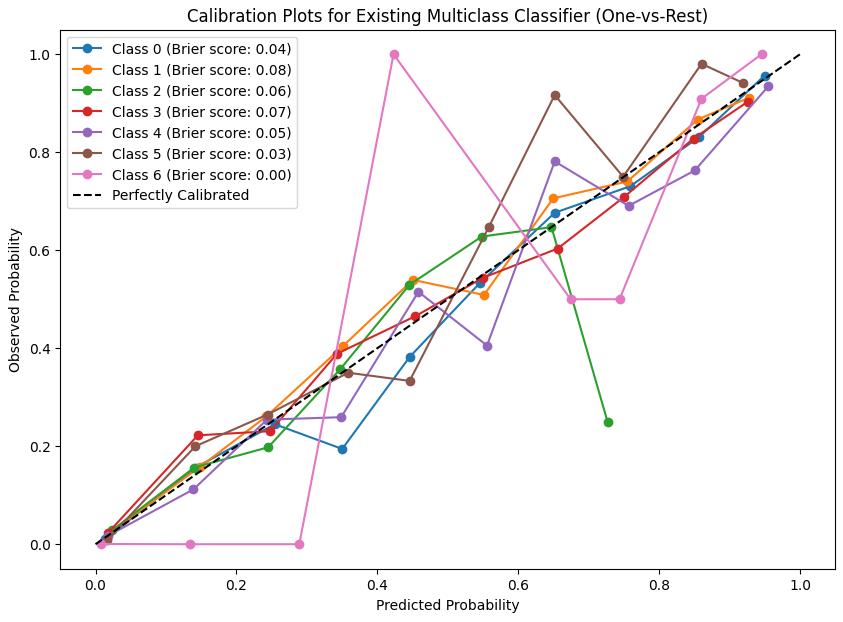


Table S1. Performance metrics of the multiclass model

| mRS score | 0 | 1 | 2 | 3 | 4 | 5 | 6 |
| --- | --- | --- | --- | --- | --- | --- | --- |
| AUC | 0.98 (0.97, 0.98) | 0.94 (0.93, 0.95) | 0.87 (0.85, 0.89) | 0.93 (0.91, 0.94) | 0.97 (0.96, 0.98) | 0.98 (0.97, 0.99) | 1.0 (1.0, 1.0) |
| NPV | 0.97 (0.96, 0.98) | 0.93 (0.92, 0.94) | 0.94 (0.93, 0.95) | 0.94 (0.94, 0.96) | 0.95 (0.95, 0.97) | 0.98 (0.97, 0.98) | 1.0 (1.0, 1.0) |
| PPV | 0.83 (0.79, 0.86) | 0.71 (0.67, 0.75) | 0.54 (0.45, 0.63) | 0.67 (0.63, 0.72) | 0.80 (0.8, 0.87) | 0.81 (0.74, 0.87) | 0.95 (0.92, 0.99) |
| Sensitivity | 0.89 (0.86, 0.92) | 0.72 (0.67, 0.76) | 0.33 (0.27, 0.4) | 0.72 (0.67, 0.76) | 0.87 (0.85, 0.9) | 0.73 (0.67, 0.8) | 0.99 (0.97, 1.0) |
| Specificity | 0.95 (0.94, 0.96) | 0.93 (0.92, 0.94) | 0.97 (0.97, 0.98) | 0.93 (0.93, 0.95) | 0.95 (0.94, 0.96) | 0.98 (0.98, 0.99) | 1.0 (1.0, 1.0) |

Figure S3. Line graph showing performance of the multiclass model across metrics and classes


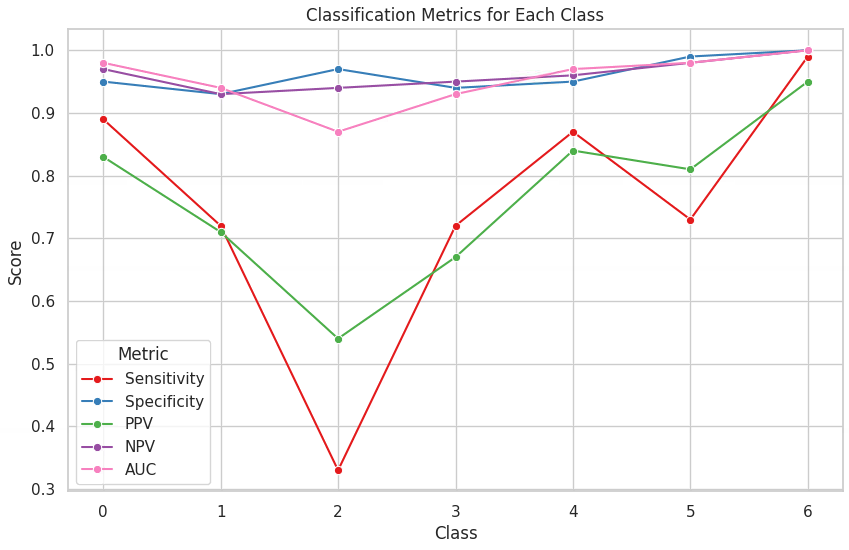


Dichotomous model:

Base model: <https://huggingface.co/UFNLP/gatortron-base>

GPU used: NVIDIA A100 Tensor Core GPU (x1)

Time to complete training: 16 hours

Training and evaluation performed in HIPAA compliant servers

Learning Rate: 1e-7

Batch size (training and evaluation): 32

Epochs: 90

Performance confidence intervals calculated based on 1,000 bootstrapped samples

Figure S4. Dichotomous model Training/validation loss curves


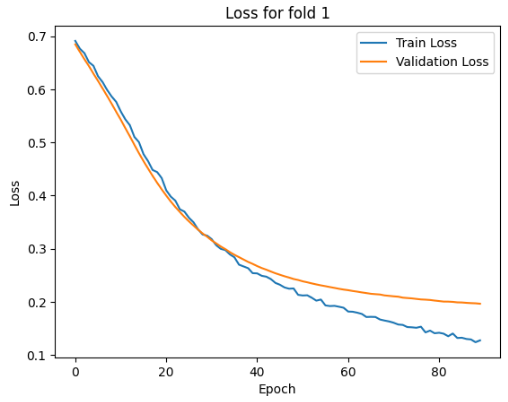

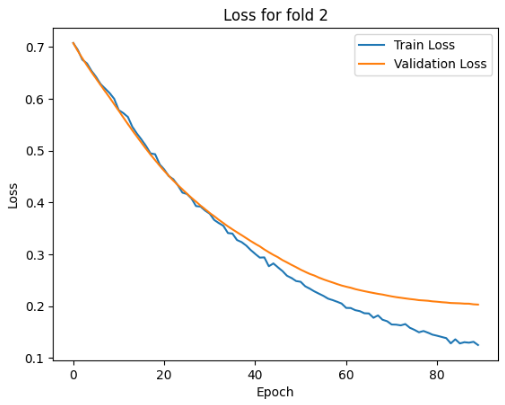

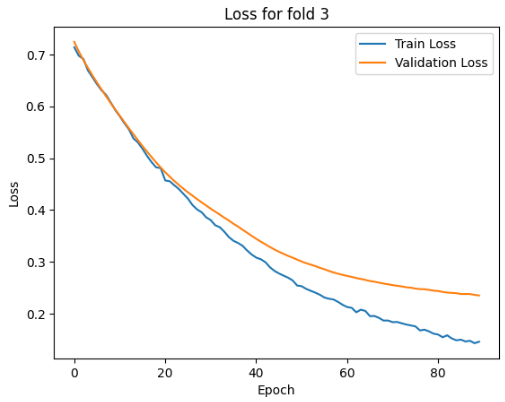

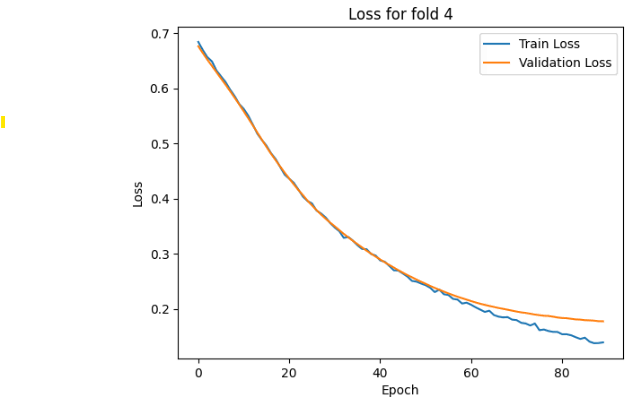


Table S2. Performance metrics of the dichotomous model

| Metric |  |
| --- | --- |
| NPV | 0.92 (0.91, 0.94) |
| PPV | 0.92 (0.90, 0.94) |
| Sensitivity | 0.92 (0.91, 0.94) |
| Specificity | 0.92 (0.91, 0.95) |
