# Supplementary material for: Assessment of the Modified Rankin Scale in Electronic Health Records With a Fine-Tuned Large Language Model: Development and Internal Validation
Source: JMIR AI. 2026 Feb 25;5:e82607. doi: 10.2196/82607 (PMC12935414; doi:10.2196/82607)
Supplement: Checklist 1 [file ai-v5-e82607-s002.docx]

Checklist 1: TRIPOD checklist.

| Section/Item | Completed Information | Page(s) |
| --- | --- | --- |
| Title (1) | Development and evaluation of prediction model for mRS using EHR | Title page (Page 1) |
| Abstract (2) | Structured abstract with objectives, methods, results, conclusion | Page 1 |
| Background 3a | Healthcare context and rationale explained | Page 2 |
| Background 3b | Target population and intended users described | Page 2 |
| Background 3c | Addressed on limitation | *Page 8* |
| Objectives (4) | Clear study objectives stated | Page 2 |
| Data 5a | Data source described (retrospective cohort from EHR) | Pages 2–3 |
| Data 5b | Data collection dates provided (Aug 2020–Jun 2023) | Page 3 |
| Participants 6a | Study setting and location described (academic hospital) | Page 3 |
| Participants 6b | Eligibility criteria for patients specified | Page 3 |
| Participants 6c | Treatments not relevant; not handled | Page 3 |
| Data Preparation (7) | Minimal preprocessing described | Pages 3–4 |
| Outcome 8a | Definition of outcome (mRS) and timing | Pages 3–4 |
| Outcome 8b | Rater qualifications described (trained mRS raters on structured cheklist) | Page 3 |
| Outcome 8c | Blinding procedures described | Page 3 |
| Predictors 9a | Predictors = EHR text passages, manually selected | Page 3 |
| Predictors 9b | Predictor assessment process detailed | Pages 3–4 |
| Predictors 9c | Patient and Raters basic demogrpahics addressed on Table 1 | *Page 12* |
| Sample Size (10) | Number of EHR passages and patients stated | Page 4 |
| Missing Data (11) | Handling of missing or discordant ratings described | Page 4 |
| Analytical Methods 12a | Analysis plan (fine-tuning LLM, cross-validation) | Pages 4–5 |
| Analytical Methods 12b | Predictor handling (raw EHR text, class weights) | Page 4 |
| Analytical Methods 12c | Model type and tuning described (fine-tuned Gatortron) | Pages 4–5 |
| Analytical Methods 12d | No heterogeneity analysis due to single-center | Implicit in Page 4 |
| Analytical Methods 12e | Performance metrics (accuracy, kappa) specified | Page 5 |
| Analytical Methods 12f | No model updating performed | Explicit on Page 5 |
| Analytical Methods 12g | Model predictions (classification) explained | Page 5 |
| Class Imbalance (13) | Addressed with weighted sampling | Page 4 |
| Fairness (14) | Fairness not evaluated; discussed as limitation | Pages 6–7 |
| Model Output (15) | Model outputs described (classification into mRS scores) | Page 5 |
| Training vs Evaluation (16) | Same data used in cross-validation folds | Page 4 |
| Ethical Approval (17) | University of Minnesota IRB approval stated | Page 3 |
| Funding (18a) | Sources of funding listed | Page 9 |
| Conflicts of Interest (18b) | No conflicts declared | Page 9 |
| Protocol (18c) | No protocol registered | *Page 6* |
| Registration (18d) | Registered at the University of Minnesota IRB | *Page 6* |
| Data Sharing (18e) | Data available upon request | Page 9 |
| Code Sharing (18f) | Code available upon request | *Page 9* |
| Patient/Public Involvement (19) | Not relevant | *Not addressed* |
| Participants Flow (20a) | Flowchart of included/excluded patients (Figure 1b) | Pages 4–5 |
| Participants Characteristics (20b) | Demographic and clinical characteristics (Table 1) | Page 8 |
| Model Development (21) | Number of samples stated | Pages 4–5 |
| Model Specification (22) | Model type and structure explained | Page 5; Appendix 1 |
| Model Performance (23a) | Accuracy and kappa reported | Pages 5–6 |
| Model Performance Heterogeneity (23b) | No heterogeneity (single-center) | Page 6 |
| Model Updating (24) | No model updating performed | Page 5 |
| Interpretation (25) | Interpretation in context of previous work | Pages 6–7 |
| Limitations (26) | Limitations discussed extensively | Pages 6–7 |
| Usability 27a | Handling missing input data discussed (selection bias) | Page 7 |
| Usability 27b | No user interaction needed post-deployment | Page 7 |
| Usability 27c | Future directions discussed | Page 7 |
